# Methodological Heterogeneity in Profilometric Assessment of Experimentally Demineralized Enamel as a Model of White Spot Lesions: A Systematic Review

**DOI:** 10.3390/jfb17090429

**Published:** 2026-08-25

**Authors:** Nadina Ilincar, Vanessa Bolchis, Mariana-Ioana Miron, Alexandru-Ionut Olari, Ramona Dumitrescu, Razvan Lacatusu, Daniela Jumanca, Atena Galuscan

**Affiliations:** 1 Translational and Experimental Clinical Research Centre in Oral Health, Faculty of Dental Medicine, “Victor Babes” University of Medicine and Pharmacy Timisoara, 300041 Timisoara, Romania; nadina.ilincar@umft.ro (N.I.); vanessa.bolchis@umft.ro (V.B.); dumitrescu.ramona@umft.ro (R.D.); razvan.lacatusu@umft.ro (R.L.); jumanca.daniela@umft.ro (D.J.); galuscan.atena@umft.ro (A.G.); 2 University Clinic of Oral Rehabilitation and Dental Emergencies, Faculty of Dentistry, “Victor Babes” University of Medicine and Pharmacy Timisoara, Eftimie Murgu Square No. 2, 300041 Timisoara, Romania; 3 Interdisciplinary Research Center for Dental Medical Research, Lasers and Innovative Technologies, Revolutiei 1989 Avenue No. 9, 300070 Timisoara, Romania; 4 Clinic of Preventive, Community Dentistry and Oral Health, Faculty of Dental Medicine, “Victor Babes” University of Medicine and Pharmacy Timisoara, 300041 Timisoara, Romania; 5 Department I, Faculty of Dental Medicine, “Victor Babeș” University of Medicine and Pharmacy Timisoara, 300041 Timisoara, Romania

**Keywords:** white spot lesions, enamel demineralization, surface roughness, profilometry, validity, reproducibility

## Abstract

Introduction: White spot lesions (WSLs) are early enamel demineralization lesions associated with increased surface roughness and plaque retention. Profilometry is commonly used to evaluate these surface changes, although methodological variability remains significant. Objective: To critically evaluate the available evidence regarding the validity and reproducibility of profilometry for assessing surface roughness in experimentally induced WSL models and treatment-modified surfaces and to identify methodological factors influencing its application across in vitro studies. Methods: A systematic review of 27 in vitro studies published between 2021 and 2026 was conducted. Methodological characteristics of contact and non-contact profilometry were synthesized, and evidence relevant to validity and reproducibility was qualitatively assessed based on complementary analytical methods and the reporting of standardized measurement procedures. Results: Considerable heterogeneity was identified in specimen sample and preparation, demineralization protocols, profilometric systems, acquisition settings, roughness parameters, and experimental conditions. Both contact and non-contact profilometry detected treatment- and demineralization-related surface changes, with Ra being the most frequent reported parameter. Complementary analytical techniques provided supporting evidence for the interpretation of profilometric findings but did not constitute formal validation. Methodological details relevant to reproducibility were inconsistently reported, and formal repeatability or reproducibility testing was generally lacking, Conclusion: Profilometry appears useful for quantitatively characterizing surface topography in experimentally demineralized enamel as a model of WSLs and treatment-modified enamel; however, substantial methodological variability and limited formal validation and reproducibility testing prevent definitive conclusions regarding its validity and reproducibility. Greater standardization of methodologies of measurement and reporting protocols is required to improve comparability and strengthen the evidence base.

## 1. Introduction

White spot lesions (WSLs) represent the earliest clinically detectable stage of enamel demineralization and remain a significant concern in both cariology and orthodontics due to their aesthetic impact and potential progression toward cavitated lesions. Their prevalence has been increasing in recent years, corresponding to a value between 10 and 49% [1]. Moreover, WSLs are a frequent finding in patients with fixed orthodontic treatments (46%) due to plaque retention caused by presence of brackets and bands [2]. Current evidence suggests that WSLs are associated with structural changes in enamel, including increased porosity and modifications of surface characteristics, particularly surface roughness. These alterations are clinically significant, as they may enhance plaque retention, facilitate lesion progression, and influence the efficacy of therapeutic interventions [1]. A WSL is defined as a localized area of enamel hypomineralization limited to the tooth surface that becomes clinically visible upon air drying [3]. Their visual appearance arises from differences in refractive indices (RI) between sound enamel (RI ≈ 1.62) and the porous lesion body, which is occupied by either water (RI ≈ 1.33) or air (RI ≈ 1.0). This disparity promotes increased light scattering, thereby producing the characteristic chalky white appearance of WSLs [4].

Recent systematic reviews indicate that despite extensive investigation into the diagnosis and management of WSLs, considerable heterogeneity remains in both diagnostic modalities and therapeutic outcomes. Moreover, no clear consensus has been established regarding the most appropriate evaluation methods or their impact on clinical decision-making [1].

Despite increasing interest in the evaluation of enamel surface roughness in WSLs, substantial methodological heterogeneity persists among experimental studies. In vitro investigations published between 2021 and 2026 show considerable variability with respect to WSL induction protocols (e.g., static demineralization vs. pH-cycling), the types of extracted human teeth employed, the timing of measurements, and the nature of post-treatment experimental challenges. For instance, some studies assessed surface roughness at baseline, post-demineralization, after treatment, and following pH-cycling, whereas others incorporated additional procedures, such as thermocycling or staining challenges, to better simulate clinical aging conditions [4,5]. Furthermore, recent evidence suggests the absence of a standardized protocol for the artificial creation of WSLs. Although pH-cycling models more closely replicate the oral environment, they also introduce increased variability and methodological complexity [6]. This variability raises concerns regarding the comparability, validity, and reproducibility of surface roughness measurements across studies.

Systematic reviews of the specialized literature have largely addressed the comparative effectiveness of therapeutic approaches for WSLs, with limited attention given to the validity of the outcome assessment methods. Thus, Lopes et al. [1] have focused on interventions such as resin infiltration, remineralization therapies, and microabrasion, primarily evaluating lesion regression or aesthetic improvement, while largely overlooking a critical appraisal of the measurement instruments used. Similarly, Puleio et al. [2], analyzing the diagnosis and management of WSLs, have either excluded in vitro studies or emphasized clinical outcomes, with limited consideration given to enamel surface characterization. More recent meta-analyses [7] have evaluated treatment efficacy using parameters such as lesion depth or microhardness, rather than focusing on surface roughness or the performance and validity of profilometric assessment methods. Consequently, there remains a clear gap in the literature regarding whether profilometry provides valid and reproducible measurements of enamel surface roughness in experimental WSL models.

The importance of addressing this gap is underscored by the increasing reliance on surface roughness as a key outcome in experimental studies. Surface roughness values vary according to the measurement technique, making the selection of an appropriate method essential. Commonly used methods for evaluating enamel alterations include contact (stylus) profilometry, non-contact optical profilometry, atomic force microscopy (AFM), and scanning electron microscopy (SEM), whereas complementary techniques such as Vickers microhardness testing are frequently employed to assess mechanical changes associated with demineralization and remineralization [8].

Profilometry is particularly relevant for the assessment of experimentally induced enamel lesions because it provides quantitative measurements of surface topography and roughness, enabling surface changes associated with demineralization and subsequent interventions to be monitored [5,9]. Both contact and non-contact approaches can be used, with the latter employing light or laser rather than a mechanical probe. Compared with complementary techniques that provide morphological information, such as scanning electron microscopy, or assess mechanical properties, such as microhardness testing, profilometry directly characterizes surface topography and roughness through numerical parameters [9]. These methods therefore provide complementary rather than interchangeable information, with profilometry being particularly relevant when treatment-related changes in enamel surface roughness are the outcome of interest. Thus, these techniques assess different characteristics of experimentally altered enamel, while profilometry is particularly relevant when surface topography and roughness constitute the outcomes of interest.

From a functional perspective, therapeutic interventions modulate enamel surface roughness through distinct underlying processes. Surface roughness is a critical factor influencing plaque retention and bacterial colonization, thereby potentially contributing to material degradation, discoloration, and the onset of gingival or periodontal inflammation [10]. Resin infiltration penetrates subsurface porosities and promotes surface homogenization, while microabrasion removes the superficial porous layer of enamel, potentially altering both roughness and thickness. Remineralizing agents, such as fluoride-based products, casein phosphopeptide-amorphous calcium phosphate (CPP-ACP), or bioactive glass, aim to redeposit minerals and reduce porosity, with variable effects on surface topography. Additionally, experimental challenges such as pH-cycling, thermocycling, or chemical staining are used to simulate clinical conditions and test the durability of treatment outcomes. Previous reviews have highlighted the heterogeneity of these interventions and their outcomes, further complicating comparisons across studies [1,2].

To the best of our knowledge, no previous systematic review has specifically examined the methodological performance, validity, and reproducibility of profilometric techniques used for enamel surface roughness assessment in experimentally induced WSLs.

In light of these considerations, a systematic review focusing specifically on the validity and reproducibility of profilometry in measuring enamel surface roughness in WSL models is warranted.

The objective of this systematic review is to critically evaluate the available evidence regarding the validity and reproducibility of profilometry for assessing enamel surface roughness in experimentally induced WSL models and subsequent treatment-modified surfaces in in vitro studies conducted on extracted human teeth. Particular emphasis is placed on methodological heterogeneity in profilometric assessment, the use of complementary surface characterization techniques, and methodological factors influencing the interpretation and reproducibility of profilometric measurements.

## 2. Materials and Methods

### 2.1. Review Question and PICO Framework

This systematic review was designed and reported in full compliance with the PRISMA (Preferred Reporting Items for Systematic Reviews and Meta-Analyses) statement, ensuring methodological transparency and rigor. The protocol was retrospectively registered in the OSF under the registration number: 10.17605/OSF.IO/23GYU. Reporting followed the PRISMA 2020 statement, and the completed checklist is provided as Supplementary Table S1.

The review question was formulated using a modified PICO(S) framework, adapted to address both measurement methodology and intervention-related changes in surface roughness:

Population (P):

Extracted human teeth with artificially induced WSLs created through in vitro demineralization protocols.

Intervention (I):

Assessment of enamel surface roughness using profilometric techniques, including both contact and non-contact profilometry, as well as two-dimensional (2D) and/or three-dimensional (3D) surface analysis systems.

Comparator (C):

Baseline measurements (sound enamel and/or post-demineralization, pre-treatment conditions).

Post-intervention measurements following therapeutic or experimental procedures (e.g., remineralization, resin infiltration, microabrasion, laser treatment, or other surface challenges such as pH-cycling or aging protocols).

Comparisons between different profilometric systems (contact vs. non-contact; 2D vs. 3D).

Comparisons with alternative surface characterization methods (e.g., atomic force microscopy [AFM], scanning electron microscopy [SEM], or other topographical assessment techniques).

Outcomes (O):

Primary outcomes:

Quantitative surface roughness parameters obtained through profilometry, including arithmetic mean roughness (Ra), root mean square roughness (Rq), maximum height of profile (Rz), arithmetical mean height (Sa), root mean square height (Sq), maximum surface height (Sz), and other reported topographical indices.

Methodological characteristics of measurement, including type of parameters reported, dimensionality (2D vs. 3D), and consistency of reporting across studies.

Indicators of validity and reproducibility of profilometric measurements (e.g., repeatability, agreement with other methods, sensitivity to experimental changes).

Secondary outcomes:

Changes in surface roughness values following post-demineralization interventions (e.g., remineralizing agents, resin infiltration, microabrasion, laser treatment).

Stability of surface roughness after experimental challenges (e.g., pH-cycling, thermocycling, staining, mechanical wear).

Study design (S):

In vitro experimental studies conducted on extracted human teeth. Accordingly, the primary research question of this review is: “Is profilometry a valid and reproducible method for measuring enamel surface roughness in artificially induced WSLs on extracted human teeth, before and after therapeutic or experimental interventions, compared with alternative surface characterization methods, in in vitro studies published between 2021 and 2026?”

### 2.2. Information Sources and Literature Research

A systematic research of the specialized literature was conducted in the following electronic databases: MEDLINE (via PubMed) and Web of Science Core Collection (Clarivate Analytics) to identify relevant studies from scientific articles published between 2021 and 2026 in each database.

The research was conducted between 1 December 2025 and 24 February 2026, with the final search performed on 24 February 2026.

In addition, ScienceDirect (via Elsevier) was searched as a supplementary source to identify potentially relevant full-text articles and publications not captured through database searching.

To ensure comprehensiveness, backward and forward citation searching was performed. The reference lists of all included studies and relevant review articles were manually screened (backward snowballing), and forward citation searching was conducted using Web of Science.

The “related articles” feature available in PubMed was also used to identify additional studies. Furthermore, Google Scholar (https://scholar.google.com, accessed on 23 February 2026) was screened as a supplementary source to identify additional potentially relevant publications, including grey literature. No additional databases or trial registers were searched.

### 2.3. Research Strategy

The research strategy was developed based on the main concepts of the review question, including WSLs, profilometry, and in vitro experimental conditions. Boolean operators (AND, OR) and truncation (*) were used where applicable to combine keywords and their synonyms.

The following research string was used as a base and adapted for each database according to its specific requirements: (“white spot lesion*” OR “demineralized enamel” OR “enamel demineralization” OR “early caries”) AND (profilometr* OR profilometry OR “surface profilometer”) AND (“in vitro” OR artificial OR laboratory).

The research syntax was modified where necessary, as some databases did not support truncation symbols or complex Boolean structures.

The research strategy was intentionally designed to be broad, without restricting terms related to specific types of profilometers or particular treatment modalities, in order to maximize sensitivity and retrieve all potentially relevant studies. Criteria related to interventions and study characteristics were applied during the screening stage.

The research of the specialized literature was limited to studies in accordance with the predefined eligibility criteria and to articles published in English. No validated search filters or automated tools were used in the development of the research strategy.

### 2.4. Eligibility Criteria

The eligibility criteria for this systematic review were defined a priori based on a modified PICO(S) framework.

Studies were included if they met the following criteria: (i) in vitro experimental studies conducted on extracted human teeth with artificially induced WSLs; (ii) assessment of enamel surface roughness using profilometric techniques (contact or non-contact; 2D and/or 3D analysis); (iii) reporting of quantitative surface roughness parameters (e.g., Ra, Rq, Rz, Sa, Sq, Sz, or equivalent); and (iv) availability of full-text articles published in peer-reviewed journals between January 2021 and February 2026, in English.

Studies were required to report (v) surface roughness measurements at least at one experimental stage (e.g., baseline, post-demineralization, post-treatment, or after experimental challenges such as pH-cycling or aging procedures). Additional (vi) surface characterization methods (e.g., SEM or AFM) were accepted only if used as complementary techniques alongside profilometry.

Studies were excluded if they (i) did not use profilometry; (ii) used only non-profilometric techniques (e.g., AFM or SEM) for surface roughness evaluation; (iii) did not report quantitative roughness parameters or provided insufficient data; (iv) were conducted on non-human substrates, clinical/in situ models, or did not involve artificially induced WSLs; or (v) were reviews, case reports, conference abstracts, or lacked full-text availability.

For synthesis, studies were grouped according to the type of intervention applied, type o profilometric method (contact vs. non-contact; 2D vs. 3D and reported roughness parameters.

For the purpose of this review, artificially induced enamel demineralization lesions corresponding to early-stage, non-cavitated WSLs were considered eligible. In vitro studies frequently describe such lesions using terms including “artificial enamel demineralization,” “initial enamel lesions,” “early caries lesions,” or “subsurface enamel lesions.” These models are widely recognized as experimental equivalents of clinical WSLs, as they reproduce the characteristic subsurface mineral loss with an intact surface layer. Therefore, studies employing these models were included, provided that the lesion involved enamel and corresponded to an early, non-cavitated stage [11,12,13].

Studies using atomic force microscopy (AFM) as the sole method for surface roughness assessment were excluded as AFM, although sometimes broadly classified within surface profilometric techniques, differs fundamentally from conventional profilometry in terms of measurement principles, spatial scale, and analyzed surface area, limiting direct comparability; therefore, AFM was considered only as a complementary technique when used alongside profilometry [14,15].

### 2.5. Selection Process

All records identified through the research strategy were exported in BibTeX format and imported into the reference management software Zotero, version 9.0.6 (64-bit) for duplicate removal. The study selection was conducted in two sequential stages. Initially, titles and abstracts of all retrieved records were screened for relevance. Subsequently, the full texts of potentially eligible studies were assessed against the predefined inclusion and exclusion criteria.

Two reviewers (NI, A-IO) independently screened each record and each retrieved report at both stages. In cases where eligibility was unclear, the full text was reviewed before a final decision was made. Disagreements regarding study eligibility were resolved through discussion until consensus was reached. The screening process was facilitated using Rayyan (https://www.rayyan.ai, accessed on 25 February 2026), a web-based platform designed to support systematic review workflows. Inter-reviewer agreement prior to consensus was high (Cohen’s κ = 0.96).

No automation tools or machine learning algorithms were used to exclude records during the screening process. No contact with study authors was required to clarify eligibility criteria. Only studies published in English were included; therefore, no translation of articles was necessary.

### 2.6. Data Extraction

Data extraction was performed independently by two reviewers (NI, A-IO) using a standardized and pre-piloted data extraction form. The data extraction form was pilot-tested on a subset of included studies and refined to ensure consistency and completeness. Extracted variables included study characteristics (author, year of publication), type of human teeth used, sample size and allocation to treatment groups, and methodological details related to artificial WSL creation.

Additionally, detailed information regarding profilometry was collected, including the type of profilometry instrument used, instrument specifications, measurement protocols, and surface roughness assessment parameters. Surface roughness outcomes were categorized according to their dimensional representation as profile-based (2D) parameters (Ra, Rq, Rz, Ry) or areal (3D) parameters (Sa, Sq, Sz, Sy). Given that these parameters characterize different aspects and dimensional representations of surface topography, they were treated as distinct outcomes.

Data on therapeutic interventions, control group characteristics, measurement timelines, study duration, and alternative therapeutic procedures were also extracted.

Furthermore, information on statistical analysis methods and extractable numerical outcomes related to surface roughness or enamel characteristics was recorded. Any discrepancies between reviewers were resolved through discussion until consensus was reached.

No assumptions were made regarding missing or unclear data. When required information was not reported, it was recorded as “not reported”.

### 2.7. Assessment of Validity and Reproducibility

Because no standardized framework currently exists for evaluating the validity and reproducibility of profilometric assessment in in vitro enamel studies, the available evidence was evaluated qualitatively using predefined criteria.

Evidence relevant to validity was identified when profilometric findings were directly compared with or accompanied by complementary analytical techniques. SEM, AFM, microhardness testing, Raman spectroscopy, and micro-CT were considered according to the specific morphological, topographical, mechanical, compositional, or substance information they provided. Findings obtained using these techniques were regarded as complementary evidence for interpreting profilometric outcomes rather than as a direct validation of profilometric measurements. Formal evidence of validity was considered to require a direct methodological comparison with an appropriate reference method under comparable experimental conditions.

Evidence relevant to reproducibility was assessed from the reporting of standardized measurement procedures, including profilometer type, calibration, acquisition settings, repeated measurements, and sufficient methodological detail to permit replication. Detailed methodological reporting was considered supportive of the assessment of reproducibility but was not interpreted as evidence of demonstrated reproducibility in the absence of formal repeatability or reproducibility testing.

### 2.8. Risk of Bias Assessment

Currently, no universally accepted risk of bias assessment tool exists specifically for in vitro studies, which justifies the use of adapted or domain-specific tools such as ROBDEMAT. The methodological quality and risk of bias of the included studies were assessed using the Risk of Bias in Dental Materials (ROBDEMAT) tool, which is specifically designed for in vitro studies in dental research [16]. The assessment was conducted independently by two reviewers (NI, A-IO).

To facilitate interpretation and consistency with widely used reporting standards, the ROBDEMAT outcomes were subsequently mapped into the Cochrane risk of bias categories (low, moderate, and high risk of bias). This approach allowed for a standardized presentation of the methodological quality across studies.

Any discrepancies between reviewers were resolved through discussion until consensus was reached. Assessment of reporting bias across studies (e.g., publication bias) was not performed due to the absence of a quantitative synthesis.

### 2.9. Data Synthesis

Quantitative synthesis was not undertaken because the structured assessment identified concurrent methodological heterogeneity across specimen characteristics, lesion-induction protocols, profilometric methodology, and intervention/post-treatment conditions, without a sufficiently comparable group of studies sharing aligned experimental conditions and roughness outcomes.

To provide a structured assessment of between-study heterogeneity, a synthesis based on the potential of variability was categorized according to studies’ methodological origin into four domains: (1) specimen-related factors, including tooth type, enamel sampling site, specimen preparation, surface standardization, and storage conditions; (2) lesion-induction-related factors, including demineralization model, solution composition, pH, duration, and cycling conditions; (3) profilometry-related factors, including measurement principle (contact or non-contact), dimensionality (2D or 3D), roughness parameters, calibration procedures, scanning protocols, and acquisition settings; and (4) intervention- and post-treatment surface-related factors, including the type of intervention, treatment procedures, subsequent experimental challenges, aging protocols, and measurement time points.

A qualitative subgroup synthesis was additionally performed according to (1) measurement principle (contact stylus-based vs. non-contact/optical profilometry) and (2) dimensional representation of the profilometric assessment (profile-based 2D vs. areal/3D assessment). Instrument capability and reported roughness outcomes were considered separately because several 3D-capable optical systems reported profile-based parameters such as Ra.

Finally, a synthesis regarding complementary analytical techniques was performed.

Given the heterogeneity in experimental protocols, interventions, roughness parameters, and measurement settings, subgroup comparisons were performed descriptively rather than quantitatively. A formal sensitivity analysis was not undertaken because no pooled quantitative effect estimate was generated. Measurement principle was classified only when explicitly reported or unambiguously described in the original article and was otherwise designated as not reported (NR).

## 3. Results

### 3.1. Study Selection

A total of 676 records were identified through database searching: PubMed (*n* = 179), Web of Science (*n* = 65), and ScienceDirect (*n* = 312). To ensure broader coverage and minimize publication bias, Google Scholar was additionally searched to identify potentially relevant literature, with the first six pages of results manually screened (*n* = 120). After the removal of duplicate entries, 395 unique records remained and were subjected to title and abstract screening. Two reviewers (NI, A-IO) independently evaluated all records against the predefined inclusion and exclusion criteria.

During title and abstract screening stage, 262 records were excluded for failing to meet the predefined inclusion criteria. The primary reasons for exclusion comprised not mentioning WSL (*n* = 181), studies involving unsuitable dental samples (*n* = 38), in vivo studies, review articles (including systematic reviews and meta-analyses), clinical trials (*n* = 29), studies utilizing inappropriate methods for the assessment of WSL (*n* = 6), and no abstract retrieved (*n* = 8). Any discrepancies in study selection were resolved through discussion until consensus was achieved.

The systematic review process was supported using Rayyan, an intelligent web-based platform designed to streamline study selection and screening. After full-text screening of the remaining 133 articles, eligibility was independently assessed. Inter-reviewer agreement prior to consensus was high (Cohen’s κ = 0.96).

Of the 133 full-text articles assessed for eligibility, 106 were excluded, primarily due to no human teeth sample (*n* = 34), no profilometer used (*n* = 26), no artificial creation of WSLs/enamel demineralization (*n* =15), no full-text available (*n* = 10), thesis (*n* = 3), outside publication date range (*n* = 3), identified duplicates (*n* = 2), not peer-reviewed (*n* = 1), in situ (*n* = 1), foreign language (*n* = 1), or using AFM (*n* = 10).

**Figure 1:**
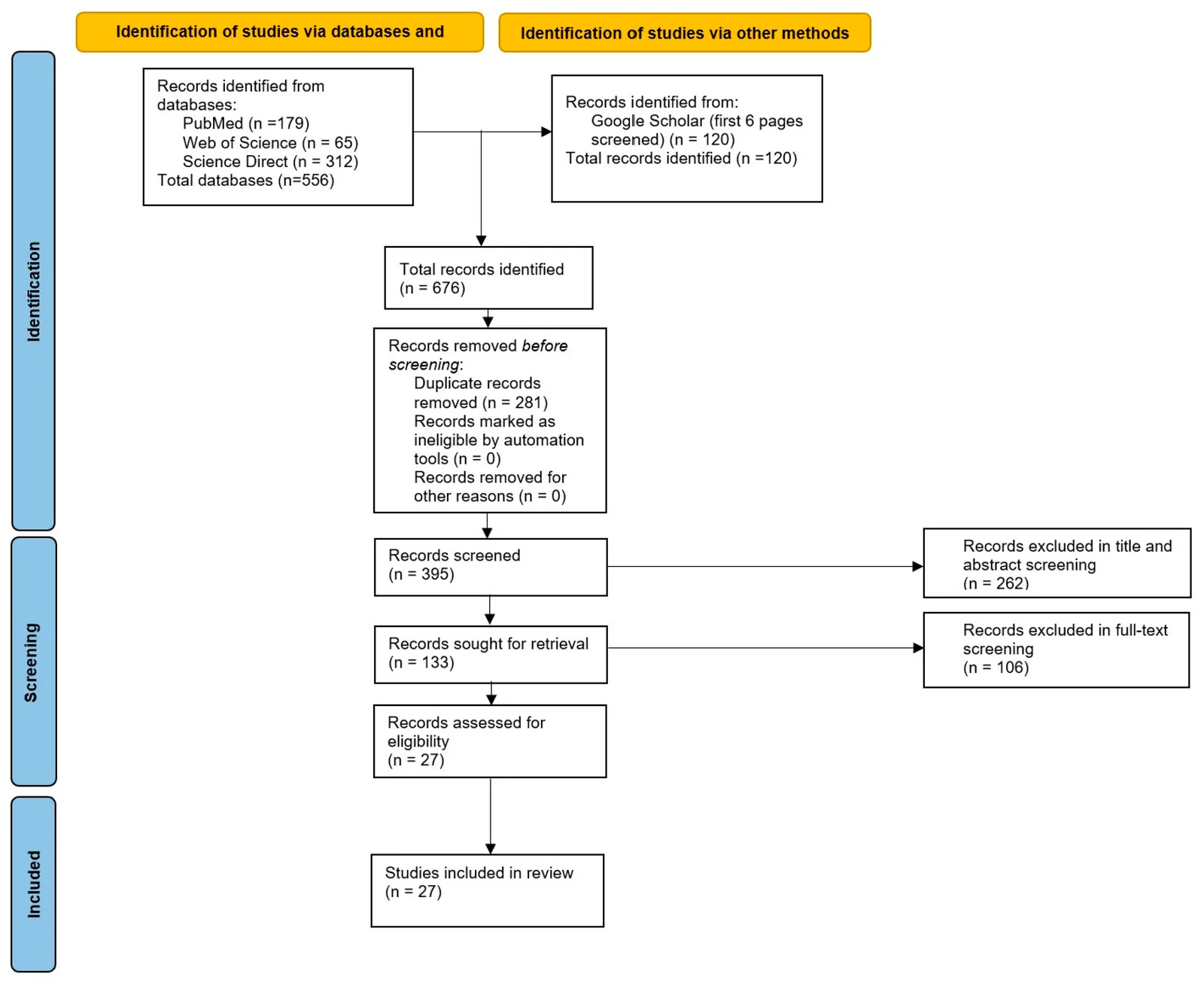
PRISMA 2020 flow diagram illustrating the study selection process [17].

Ultimately, 27 studies were included in the systematic review. The study selection process is shown in Figure 1.

Several studies that initially appeared to meet the inclusion criteria were excluded following full-text assessment. The primary reasons for exclusion included the absence of human teeth samples, lack of profilometric assessment, and failure to involve artificially induced WSLs or enamel demineralization.

For example, studies by authors Karadeniz et al. [18] and Sinanovic et al. [19] were excluded due to the use of non-human samples, while Manso et al. [20] did not employ profilometry as an outcome assessment method. Additionally, the study by Shah, P. et al. [21] was excluded because no artificial WSL model was used.

### 3.2. Study Characteristics

A total of 27 in vitro studies were included in this systematic review. All studies were conducted on extracted human teeth, although variability was observed in the type of teeth used, including premolars, molars, and primary teeth. Sample sizes and allocation to experimental groups varied considerably across studies.

Artificial WSLs were induced using different experimental protocols. The most commonly employed methods included pH-cycling models and static demineralization procedures, although variations in duration, solution composition, and experimental conditions were noted.

Considerable heterogeneity was also observed in profilometric assessment methods. Both contact (stylus-based) and non-contact (optical) profilometry techniques were used, with studies reporting either profile-based (2D) or areal (3D) surface roughness parameters.

The included studies investigated a wide range of therapeutic interventions, including resin infiltration, fluoride-based and remineralizing agents, nano-materials, laser-based treatments, dentifrices, and surface treatment methods such as microabrasion or air polishing. A detailed summary of the characteristics of the included studies is presented in Table 1.

### 3.3. Risk of Bias Assessment

The risk of bias assessment revealed variability in methodological quality across the included studies.

**Figure 2:**
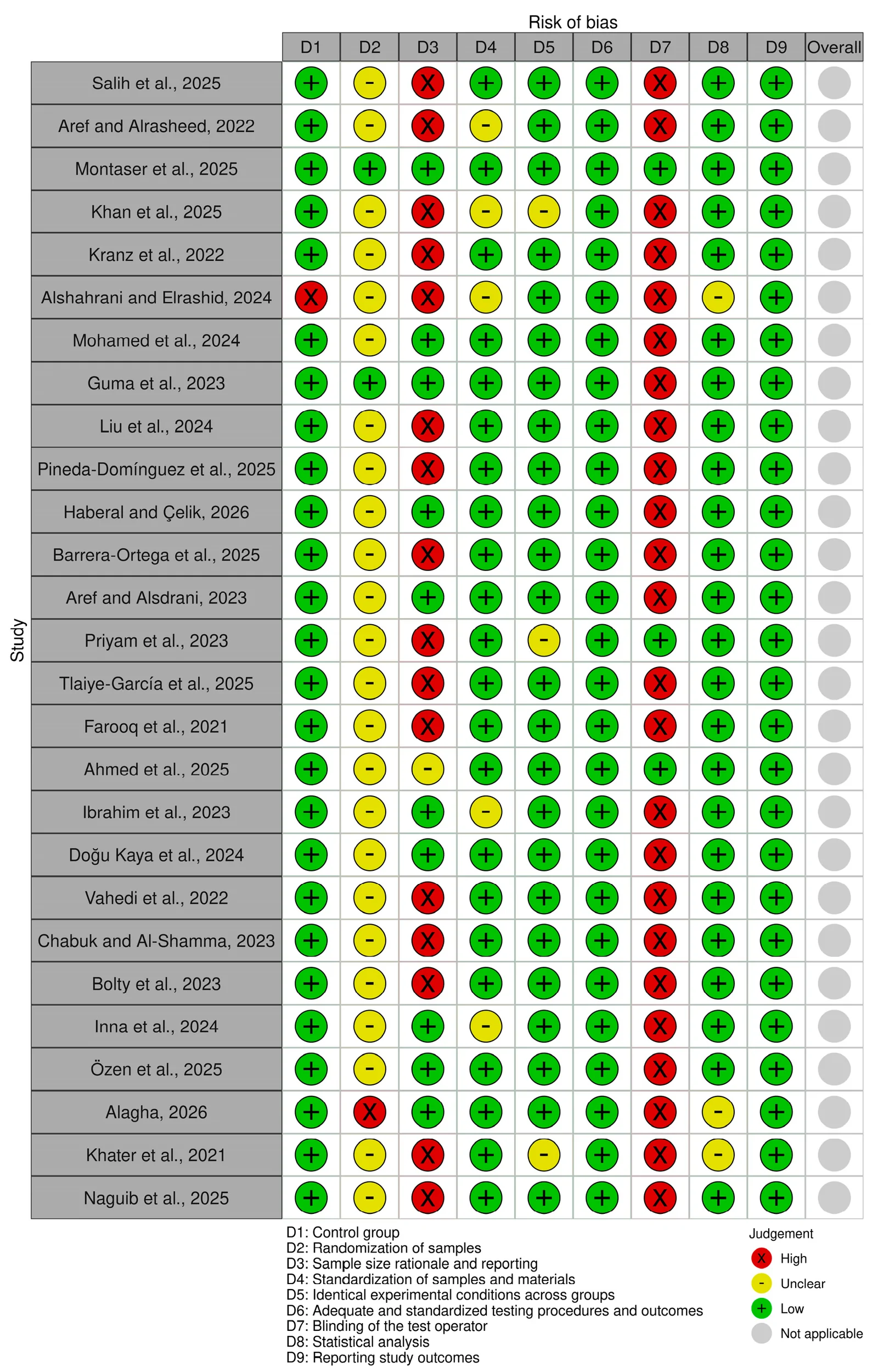
Risk of bias assessment.

However, recurrent methodological limitations were identified, particularly in domains related to specimen randomization blinding of the test operator and sample size calculation. The RoBDEMAT assessment identified recurrent reporting concerns, particularly for randomization, sample-size rationale, and operator blinding. Randomization was sufficiently reported in only 3 of 27 studies, while it was insufficiently reported in 23 and not reported in one. Sample-size rationale was sufficiently reported in 10 studies, insufficiently reported in two, and not reported in 15. Operator blinding represented the most frequent concern, being not reported in 22 studies and not adequate in two, while only three studies provided adequate or sufficient reporting. In contrast, specimen standardization and identical experimental conditions were generally better documented (22/27 and 24/27 studies, respectively). Testing procedures and outcome assessment were adequately or sufficiently reported in all studies, while statistical analysis was adequately or sufficiently reported in 24/27 studies. Overall, the most recurrent RoBDEMAT concerns were related to randomization, sample-size justification, and operator blinding. Overall, recurrent methodological concerns were identified, particularly regarding operator blinding and sample size justification, while randomization procedures were frequently insufficiently reported. In contrast, outcome measurement and reporting domains generally showed a lower risk of bias. Overall, the included studies were considered to present a moderate risk of bias, primarily due to incomplete reporting rather than fundamental flaws in experimental design. The detailed risk of bias assessment is presented in Figure 2.

### 3.4. Results of Individual Studies

All 27 included studies were in vitro experimental investigations conducted on extracted human teeth or enamel specimens derived from them. The studies evaluated artificially demineralized enamel using different specimen configurations and therapeutic or experimental interventions, including remineralizing agents, resin infiltration, microabrasion, laser-assisted treatments, bleaching, toothbrushing, and air polishing. Profilometric assessment was performed at different experimental stages, with some studies additionally incorporating post-treatment challenges such as pH cycling, thermocycling, chemical aging, or staining. Detailed characteristics of the individual studies are presented in Table 1.

All included studies reported quantitative outcomes related to enamel surface roughness, expressed using different profile-based and areal parameters. The most frequently reported profile-based parameters were Ra and Rz, whereas areal assessments included Sa.

In addition to profilometric measurements, many studies reported complementary outcomes, most frequently surface microhardness [41], morphological changes assessed by scanning electron microscopy [29,46], and mineral composition analyzed using techniques such as energy-dispersive X-ray spectroscopy (EDX) [26] or Raman spectroscopy [31].

### 3.5. Results of Syntheses

Due to the substantial heterogeneity in study design, intervention protocols, and outcome assessment methods, a narrative synthesis was conducted. Type of profilometric method (contact vs. non-contact; 2D vs. 3D) and reported roughness parameters are provided in Table 2.

Ra was the predominant roughness outcome, being explicitly reported in 21 of the 27 included studies. Rz was additionally reported by Tlaiye-García et al. and Vahedi et al., while Haberal and Çelik reported both Ra and the areal parameter Sa [11,35,40]. Alagha reported Ry, whereas five studies described the outcome only as surface roughness without specifying the exact roughness parameter [43].

#### 3.5.1. Subgroup Synthesis According to Sources of Heterogeneity Among Studies

##### Specimen-Related Heterogeneity

Specimen-related heterogeneity was characterized according to dentition, tooth type, specimen configuration, surface preparation, and pre-experimental storage.

Of the 27 included studies, 25 used permanent teeth, whereas two used deciduous anterior teeth. Montaser et al. used exfoliated primary anterior teeth, while Tlaiye-García et al. investigated naturally extracted or exfoliated anterior deciduous teeth [24,35].

Among the permanent-tooth studies, premolars were the exclusive substrate in 10 studies [4,22,23,25,27,36,37,38,41,42], six used molars [5,29,30,33,39,45], and five specifically used third molars [11,26,31,32,40]. Two studies used permanent anterior teeth [34,43], one included both molars and premolars [28], and one reported teeth obtained from orthodontic extractions without specifying tooth type [44].

Specimen preparation followed several recurring configurations rather than a single protocol. Some studies retained the crown or a large crown segment and exposed a defined enamel window. Montaser et al., for example, retained a 3 × 4 mm labial enamel window and fixed the teeth in self-curing acrylic, whereas Alshahrani and Elrashid embedded the separated coronal portion in acrylic and assessed the central buccal region [24,27]. Other studies generated discrete enamel specimens. Liu et al. prepared four blocks from each molar and polished them sequentially with 500-, 1200-, 2500-, and 4000-grit SiC paper to obtain flat 6 × 4 mm surfaces [30]; Farooq et al. produced approximately 5 × 5 mm acrylic-embedded enamel blocks and polished the exposed enamel with 1200-grit abrasive paper [36]; and Salih et al. prepared 4 × 4 mm buccal and lingual enamel pieces, embedded them in acrylic, and polished the surfaces sequentially with P800, P1200, and P2000 papers [22]. Guma et al. similarly used a standardized sequential 800/1200/4000-grit polishing protocol before profilometric assessment [29]. In contrast, other studies retained anatomically curved enamel surfaces or defined experimental windows without an equivalent flattening sequence [24,35,38].

Pre-experimental storage also showed recurring but non-identical protocols. Thymol-based storage was the most frequent approach, being explicitly reported in 14 of the 27 studies [4,5,11,22,23,24,25,27,30,33,35,37,38,42]. Concentration and temperature nevertheless differed: Montaser et al. and Tlaiye-García et al. used 0.2% thymol, the latter at 4 °C [24,35]; Ibrahim et al. used 0.1% thymol at room temperature [38]; Haberal and Çelik used 0.1% thymol at +4 °C [11]; Liu et al. used 0.1% thymol until use [30]; and Ahmed et al. combined 70/30 ethanol disinfection with subsequent refrigerated storage in 0.5% thymol [37]. Khan et al. likewise reported storage in 0.1% thymol until measurement [25].

Other pre-experimental media included distilled/deionized water in four studies, artificial saliva in three, formaldehyde/formalin-based disinfection or storage in two, and chloramine-T followed by artificial-saliva storage in one study. Guma et al. stored molars in distilled water at 25 °C [29], Doğu Kaya et al. stored extracted molars in distilled water before preparing the enamel blocks [39], and Farooq et al. maintained the prepared enamel blocks in artificial saliva before, during, and after the experimental procedures [36]. Alagha similarly stored the separated crowns in artificial saliva at room temperature [43]. Naguib et al. disinfected specimens in 10% neutral phosphate-buffered formalin for five days [45], whereas Mohamed et al. kept specimens in 2% formaldehyde at room temperature until use [28]. Vahedi et al. used 0.05% chloramine-T for disinfection followed by artificial saliva at 27 °C [40]. For the remaining studies, a directly comparable pre-experimental storage protocol was not sufficiently specified.

##### Lesion-Induction-Related Heterogeneity

Lesion-induction protocols were classified according to the principal demineralization model, solution composition, pH, exposure duration, and use of subsequent pH cycling. A discrete static demineralization phase before treatment was used in 22 of the 27 studies [4,5,11,22,23,24,25,26,27,28,30,31,32,33,34,37,38,39,41,42,43,45]. Three studies used a pH-cycling model as the principal lesion-induction procedure [29,40,44], while two studies employed predominantly erosive rather than conventional caries-like challenges [35,36]. Guma et al., for example, generated demineralized enamel through a two-week pH-cycling protocol, while Khater et al. evaluated enamel before and after lesion induction by pH cycling [29,44]. Farooq et al. instead used 6 wt.% citric acid at pH 2.4 for 5 min, whereas Tlaiye-García et al. exposed deciduous enamel to a laboratory demineralizing solution or acidic beverages for 4 and 7 days [35,36].

Among the static caries-like models, calcium-phosphate solutions buffered with acetic acid or acetate represented the most recurrent formulation, although concentrations and experimental conditions differed among studies. Liu et al. used 2.0 mmol/L calcium, 2.0 mmol/L phosphate, and 0.075 mol/L acetate at pH 4.5 for 48 h at 37 °C, whereas Mohamed et al. used 2.2 mM calcium nitrate, 2.2 mM potassium phosphate, and 50 mM acetic acid at pH 4.2–4.25 for 3 days [28,30]. Montaser et al. used 2.2 mM CaCl_2_, 2.2 mM KH_2_PO_4_, and 0.05 M acetic acid at pH 4.4 for 96 h, while Naguib et al. used 2.2 mM CaCl_2_, 2.2 mM KH_2_PO_4_, and 50 mM acetic acid at approximately pH 4.2–4.5 for 7 days at 37 °C, with daily solution replacement [24,45]. Ibrahim et al. similarly used a calcium/phosphate/acetic-acid system at pH 4.4 but maintained specimens for 7 days at 37 °C [38].

Other static protocols used different acidic systems. Inna et al. induced WSLs for one week at 37 °C using 6% hydroxyethyl cellulose and 0.1 M lactic acid at pH 4.5, while Chabuk and Al-Shamma used a lactic-acid-based demineralization protocol [4,5]. The duration of the initial lesion-induction phase therefore ranged from 48 h in Liu et al. to 3 days in Mohamed et al. and Bolty et al. [28,30,41]; 4 days/96 h in Khan et al., Montaser et al., Doğu Kaya et al., and Ahmed et al. [24,25,37,39]; and 7 days in Ibrahim et al., Inna et al., and Naguib et al. [4,38,45].

pH cycling was incorporated in 12 of the 27 studies, either as the primary lesion-induction procedure or as a subsequent demineralization-remineralization challenge [5,24,28,29,30,31,32,37,38,40,41,44]. The cycling schedules were not identical. Montaser et al. used a 28-day regimen including a 2-h daily acid challenge, Liu et al. used 6 h of demineralization at pH 4.7 followed by 18 h of remineralization at pH 7.0 for 5 days, Mohamed et al. used 2 h of demineralization followed by 22 h of remineralization daily for one month, and Ahmed et al. subjected treated specimens to a 14-day pH-cycle challenge [24,28,30,37].

Static chemical demineralization represented the predominant lesion-induction approach, being used in 22 of the 27 included studies, most commonly through calcium-phosphate solutions buffered with acetic acid or acetate. pH-cycling protocols were also frequently incorporated, being used in 12 studies either for primary lesion induction or as a subsequent demineralization-remineralization challenge.

##### Profilometry-Related Heterogeneity

Profilometry-related heterogeneity was characterized according to measurement principle, instrument configuration, dimensional representation, roughness parameter, surface-sampling strategy, calibration, and acquisition settings. As detailed in the methodological subgroup analysis, 11 of the 27 studies used contact-based profilometry, 11 used non-contact/optical systems, 1 employed both approaches [26], and 4 did not provide sufficient information for classification [22,25,34,37].

Considerable instrument-level diversity was present within both measurement categories. Contact measurements were performed using different stylus-based systems, including Mitutoyo/Surftest, Surtronic, MarSurf, TR200, DEKTAK, and Ambios instruments [23,33,38,40,42,44]. For example, Aref and Alrasheed used a Mitutoyo contact profilometer with a 2-µm-radius, 60° diamond stylus, 0.75-mN measuring force, 3-mm traverse, and three measurements per specimen [23]. Aref and Alsdrani used a Surftest 211 with a 6-mm measuring distance, 0.5-mm/s measuring speed, 0.75-mN force, and a 2-µm/60° stylus, with measurements obtained at five locations per specimen [33]. Priyam et al. reported the use of a Surtronic S128 profilometer and instrument calibration according to the manufacturer’s instructions but did not provide sufficient information in the article to classify the measurement principle [34].

The non-contact group likewise comprised different optical measurement principles and instrument configurations, including white-light interferometric, confocal white-light, and other 3D optical systems. Pineda-Domínguez et al. used a ZYGO Nexview 3D optical profilometer and obtained six randomly positioned Ra measurements from each 3 × 6 mm^2^ enamel surface; representative 3D images were acquired at 100× over an 83.139 × 83.139 µm^2^ area [31]. Haberal and Çelik used a Profilm 3D white-light interferometric system to evaluate a central 2 × 2 mm area with a reported sensitivity of 0.05 µm and calculated both Ra and Sa [11]. Barrera-Ortega et al. used a ZYGO 3D-Nexview system and recorded six randomly positioned Ra measurements on a 3 × 6 mm^2^ working surface at several experimental time points [32].

Roughness outcome selection was dominated by Ra, which was explicitly reported in 21 of the 27 studies. Additional roughness descriptors were less frequently reported: Rz was reported in two included studies [35,40], Sa was reported together with Ra by Haberal and Çelik [11], and Ry was reported by Alagha [43]. Five studies reported surface roughness without clearly specifying the corresponding roughness parameter [22,25,27,28,41]. Thus, the evidence base predominantly consisted of profile-average roughness measurements, with limited use of additional profile- or area-based descriptors.

Surface-sampling protocols also differed in the number, location, and spatial distribution of measurements. Aref and Alrasheed obtained three measurements per specimen, whereas Aref and Alsdrani obtained measurements at five different locations [23,33]. Pineda-Domínguez et al. and Barrera-Ortega et al. each used six randomly positioned Ra measurements over a defined enamel surface [31,32]. Other protocols used predefined linear traces or surface areas, and the number and spatial arrangement of measurements were not uniformly reported across all studies.

Calibration or calibrated instrument status was explicitly mentioned in four studies [5,33,34,42], although the level of detail regarding the calibration procedure, reference standard, or frequency varied. Stylus or probe characteristics, measuring force, scan length or area, scanning speed, cut-off or filtering procedures, resolution, measurement location, and software or data-processing settings were inconsistently documented. Some studies provided relatively detailed acquisition protocols [23,33,42], whereas others omitted several characteristics relevant to complete reconstruction of the measurement procedure [24,31,36,39]. In four studies (Salih et al.; Khan et al.; Priyam et al.; Ahmed et al.) [22,25,34,37], the reported information was insufficient to classify the measurement principle as contact-based or non-contact/optical.

Accordingly, the most recurrent profilometry-related patterns across the 27 studies were the predominance of Ra as the reported roughness outcome (21/27), the balanced representation of classifiable contact and non-contact/optical measurement principles (11 studies each), and limited detailed reporting of calibration procedures. Measurement number and spatial distribution, stylus/probe characteristics, measuring force, scan length or area, speed, cut-off/filtering, resolution, and software or data-processing settings were reported to varying extents across the included studies.

##### Intervention- and Post-Treatment Surface-Related Heterogeneity

Intervention- and post-treatment surface-related heterogeneity was characterized according to treatment category, treatment combinations, post-treatment experimental challenges, and the stage at which profilometric measurements were obtained. The interventions were highly diverse, and several studies evaluated more than one treatment category; therefore, the categories were not mutually exclusive.

Resin infiltration or other resin-based surface treatments were investigated in 12 of the 27 studies [4,5,23,25,27,30,33,37,38,39,42,43]. These protocols included conventional ICON infiltration, modified etching/infiltration procedures, pit-and-fissure sealants, universal adhesives, nano-hydroxyapatite-containing adhesive resins, bioactive-glass-modified experimental infiltrants, and combinations of resin infiltration with remineralizing agents. Aref and Alrasheed, for example, compared ICON, CPP-ACP, universal adhesive resin, and sequential CPP-ACP/universal adhesive treatment, whereas Ahmed et al. investigated experimental TEGDMA/UDMA resin infiltrants containing three different bioactive glasses [23,37].

Remineralization-oriented interventions included fluoride- and calcium/phosphate-containing varnishes and pastes, CPP-ACP/CPP-ACP-F, nano-hydroxyapatite-containing systems, zinc-carbonate hydroxyapatite, fluoridated bioactive-glass formulations, nanosilver fluoride, natural remineralizing agents, and MgO nanoparticle-containing formulations. Treatment schedules ranged from single or short-term applications to repeated applications over several days or weeks. Barrera-Ortega et al., for example, evaluated β-TCP-F and CPP-ACP-F over 5, 10, and 15 days, while Naguib et al. assessed six remineralization conditions containing conventional agents and/or MgO nanoparticles at 2, 4, and 8 weeks [32,45].

Dentifrice/brushing-based interventions were the principal experimental treatment in four studies [24,26,34,36], laser-based treatment was investigated in two studies [22,40], microabrasion in two studies [4,5], and air polishing or bioactive-glass air-abrasion in two [5,29]. Farooq et al. used 5000 brushing strokes with either fluoride or fluoridated bioactive-glass toothpaste, while Guma et al. evaluated erythritol and sodium-bicarbonate air polishing after 5 and 150 s of exposure [29,36].

Post-treatment profilometric assessment also differed according to whether an additional experimental challenge was applied after the intervention. A distinct post-treatment pH-cycling challenge followed by roughness assessment was used in four studies [5,28,30,41]. Chabuk and Al-Shamma measured roughness at baseline, after demineralization, after treatment, and after a final pH-cycling stage, while Bolty et al. assessed specimens before and after treatment and again following pH cycling [5,41]. Mohamed et al. applied NSF or NaF varnish before a 30-day pH-cycling protocol, with surface roughness evaluated at the end of the experimental period [28]. In contrast, in studies such as Barrera-Ortega et al. and Pineda-Domínguez et al., pH cycling formed part of the ongoing remineralization protocol itself, with repeated assessments at 5, 10, and 15 days, rather than constituting a separate challenge after completion of treatment [31,32].

Thermocycling followed by profilometric reassessment was used in four studies [4,30,37,42]. Ahmed et al. used 5000 thermal cycles and additionally performed chemical aging, with surface roughness recorded before and after both procedures, whereas Özen et al. recorded Ra at baseline, after demineralization, after treatment, and following 5000 thermal cycles [37,42]. Inna et al. combined 5000 thermal cycles with red-wine exposure and compared these specimens with controls stored in artificial saliva, while Liu et al. separately subjected resin-infiltrated specimens to four simulated long-term challenges—pH cycling, thermocycling, staining, and toothbrushing—with roughness measured before and after each challenge [4,30].

Other post-treatment conditions were less frequently represented. Bleaching after resin infiltration was assessed in two studies [27,43], simulated post-infiltration brushing in two [30,43], staining exposure in two [4,30], chemical aging in one [37], and post-infiltration polishing in one [43]. Alshahrani and Elrashid measured roughness after different resin-infiltration modalities and again after subsequent bleaching, whereas Alagha compared brushing, bleaching, and polishing after ICON treatment and evaluated specimens after one day, one week, and one month of storage [27,43]. Follow-up without an additional active aging challenge was also performed by Ibrahim et al., who repeated profilometric measurements at 7 and 28 days after completion of resin infiltration [38]. Tlaiye-García et al. represented a separate experimental model in which CPP-ACP fluoride-varnish-treated deciduous enamel was subsequently exposed to different acidic media and evaluated after 4 and 7 days [35].

Overall, profilometric assessment was therefore performed on surfaces representing different experimental states: immediately after treatment, during repeated remineralizing treatment, after defined follow-up periods, or after subsequent chemical, thermal, mechanical, or erosive challenges.

#### 3.5.2. Overall Methodological Trends and Reporting Patterns

Across the 27 included studies, several recurring methodological trends and reporting deficiencies were identified. Profilometry was widely used for quantitative assessment of experimentally demineralized and intervention-modified enamel, with contact and non-contact/optical approaches equally represented among studies for which the measurement principle could be classified (11 studies each), while Ra was the predominant roughness outcome (21/27 studies). Static chemical demineralization was the most frequently used lesion-induction approach (22/27 studies), although experimental protocols varied across studies. Repeated profilometric measurements within assessment stages or across experimental time points were also commonly performed. In contrast, several technical aspects relevant to methodological replication were inconsistently reported. Calibration or calibrated instrument status was explicitly mentioned in four studies, although the level of detail regarding the calibration procedure, reference standard, or frequency varied. Measurement number and spatial distribution, stylus/probe characteristics, measuring force, scan length or area, resolution, cut-off/filtering, and software or data-processing settings were also reported to varying extents. Thus, the most consistent pattern across the evidence base was the widespread use of quantitative profilometry accompanied by limited standardization and incomplete reporting of key measurement procedures.

#### 3.5.3. Subgroup Synthesis According to Profilometric Methodology and Dimensional Representation of the Profilometric Assessment

##### Subgroup Synthesis According to Profilometric Methodology

When stratified according to measurement principle, explicitly reported, or unambiguously described in the original articles, 11 studies used contact-based profilometry [5,23,28,33,35,38,39,40,42,44,45], while 11 used non-contact/optical profilometry [4,11,24,27,29,30,31,32,36,41,43]. One study employed both approaches on different dental substrates [26], and four provided insufficient information for classification [22,25,34,37].

Both methodological groups detected surface roughness changes across experimental stages, but the available data were not sufficiently comparable for quantitative pooling or direct numerical comparison between contact and optical approaches. Direct within-study comparison was also limited: Kranz et al. employed both white-light interferometric and mechanical profilometry, but the latter was applied to dentin rather than enabling a standardized comparison on the same enamel surface [26]. Accordingly, no consistent between-subgroup pattern could be identified that differentiated contact from non-contact/optical profilometry on the basis of the reported roughness outcomes.

##### Subgroup Synthesis According to Dimensionality

Subgrouping according to dimensionality revealed a distinction between instrument capability and the dimensional representation of the reported roughness outcome. Explicit profile-based 2D assessment was reported, for example, by Guma et al., Chabuk and Al-Shamma, and Özen et al. [5,29,42], whereas explicitly 3D-capable optical systems were used by Kranz et al., Alshahrani and Elrashid, Pineda-Domínguez et al., Haberal and Çelik, Barrera-Ortega et al., and Bolty et al. [11,26,27,31,32,41]. However, 3D instrument capability did not consistently correspond to areal roughness reporting. Kranz et al. generated profile-based Ra values from 3D optical data, and Pineda-Domínguez et al. similarly reported Ra despite using a 3D optical system [26,31]. Haberal and Çelik reported both Ra from linear scans and Sa over the scanned surface [11].

Overall, profile-based Ra remained the predominant quantitative outcome across both contact and optical systems, whereas explicitly reported areal parameters were less frequent.

#### 3.5.4. Complementary Techniques Synthesis

Complementary analytical techniques were used in several included studies to characterize aspects of the experimental substrate not captured by profilometric roughness measurements alone. SEM was used to evaluate surface morphology and treatment-associated structural alterations in eight studies [11,23,29,32,33,37,42,45]. Raman spectroscopy was employed by three studies [26,31,44] to assess mineral- and composition-related characteristics. Micro-CT was used in three studies [36,37,42] to characterize subsurface or structural characteristics of the experimental enamel. Microhardness testing and elemental analyses such as EDX were also incorporated in several studies as complementary mechanical and compositional assessments. In contrast, AFM was not identified as a complementary measurement technique among the included studies. None of these complementary techniques were applied as a formal reference standard for direct validation of profilometric roughness measurements.

### 3.6. Reporting Biases

Formal assessment of publication or reporting bias was not conducted due to the considerable methodological heterogeneity of the included studies and the lack of quantitative synthesis. Conventional approaches used for publication bias evaluation, such as funnel plot analysis or related statistical tests, were considered unsuitable given the variability in study designs, therapeutic interventions, profilometric methodologies, and reported outcomes. However, several measures were implemented to reduce the potential risk of reporting bias, including comprehensive electronic database searching, manual screening of reference lists, forward citation tracking, and supplementary literature searches performed through Google Scholar.

## 4. Discussion

Among the methodological domains examined, profilometry-related factors were most directly associated with the acquisition and numerical representation of surface roughness. In the present review, among studies with a classifiable measurement principle, contact and non-contact/optical approaches were equally represented (11 studies each), while Ra predominated (21/27 studies), despite substantial variation in instrumentation, sampling strategies, acquisition settings, and calibration reporting.

Lesion-induction protocols represented a second major source of variability, as static chemical demineralization predominated (22/27 studies), but solution composition, pH, exposure duration, temperature, and pH-cycling conditions differed. Experimental evidence from Billoo et al. on bovine enamel suggests that variations in pH-cycling conditions may result in differences in surface roughness, lesion depth, mineral loss, and hardness [47,48]. These findings suggest that differences in lesion-induction protocols may contribute to variability in the characteristics of experimentally produced enamel lesions before profilometric assessment.

The predominance of static demineralization models suggests a general preference for controlled lesion-induction conditions across the included studies. Nevertheless, conventional pH cycling was also incorporated in nearly half of the studies (13/27), either during lesion formation or after treatment. The considerable variation in cycling schedules, including differences in the duration and frequency of demineralization and remineralization phases, represents an additional source of methodological heterogeneity.

The use of static caries-like models alongside cyclic demineralization-remineralization protocols and more aggressive erosive or acid-etching approaches further illustrates the variability in lesion-induction methodology. Such differences may influence the characteristics of the resulting enamel lesions and should therefore be considered when comparing profilometric surface roughness findings across studies.

Intervention and post-treatment conditions introduced an additional level of variability, because profilometry was performed after fundamentally different surface-modifying treatments and at different experimental stages, ranging from immediate post-treatment assessment to measurements following pH cycling, thermocycling, brushing, staining, chemical aging, or storage. These therefore represent different post-treatment surface states rather than a uniform experimental endpoint.

Finally, specimen-related characteristics represented an additional potential source of pre-analytical variability. The included studies differed in enamel origin and tooth type, surface preparation, and storage conditions, which may have contributed to differences in the experimental substrate before lesion induction and profilometric assessment. Evidence from extracted-tooth research indicates that storage conditions can influence in vitro experimental outcomes, with the magnitude and direction of these effects varying according to the dental substrate, storage protocol, and outcome evaluated [48]. However, because specimen-related factors varied concurrently with other methodological domains in the included studies, their specific contribution to the reported roughness values could not be determined.

Overall, profilometry-related factors were the most proximal to the measured roughness outcome, followed by lesion-induction and intervention/post-treatment conditions, whereas specimen-related factors represented a more indirect source of variability. However, because these domains generally varied concurrently across the included studies, their independent contributions could not be disentangled or quantitatively ranked. Therefore, no single predominant source of heterogeneity could be established from the available evidence.

This methodological distinction is particularly relevant when comparing contact and non-contact profilometry, which rely on fundamentally different surface-sampling mechanisms. In the included studies by Aref and Alrasheed, Mohamed et al., Chabuk and Al-Shamma, and Özen et al., contact profilometry was predominantly based on stylus tracing and profile-derived parameters such as Ra, with measurement conditions varying in stylus radius, tip angle, measuring force, traverse length, cut-off, and scanning speed [5,23,28,42]. These parameters are metrologically relevant because the finite stylus geometry acts as a mechanical spatial filter: sharp valleys or closely spaced surface features may not be fully accessed by the probe, and changes in stylus radius can systematically alter measured roughness values [49].

Tracing speed, measuring force, evaluation length, and filtering represent additional methodological parameters that varied across the included studies. Among these factors, experimental metrological evidence has demonstrated that changes in stylus tracing speed can influence the resulting roughness measurements [50].

Conversely, the optical systems identified in the present review included white-light interferometry, confocal white-light sensing, and other 3D non-contact approaches, with substantial variation in scan area, lateral/vertical resolution, magnification, sampling density, and data-processing procedures [11,26,29,31]. Optical systems acquire surface topography without mechanical contact and can provide areal surface characterization; however, their metrological performance depends on the measurement principle, instrument characteristics, acquisition conditions, and calibration. These aspects are addressed within the ISO 25178 framework for areal surface texture, which defines metrological characteristics of areal topography measuring methods and procedures for their calibration and verification [51]. Recent metrological research has further emphasized that these characteristics contribute to measurement uncertainty in optical areal surface measurements and should therefore be considered when comparing measurements obtained using different instrument configurations [52].

Together with the measurement principle and acquisition-related effects discussed above, Vorburger et al. and Rosentritt et al. suggest that differences in roughness magnitude across studies may reflect both actual surface variation and methodological differences in how surface topography is sampled and represented [53,54]. Cross-study differences in roughness values should therefore be interpreted cautiously, as they may not reflect differences in the experimental surface alone. Direct comparative evidence further shows that the measurement principle can affect reported roughness values, with discrepancies observed between stylus and white-light interferometric measurements [53] and between contact and optical measurements of Ra/Sa and Rz/Sz in dental materials [54].

Differences in roughness values reported under apparently similar experimental conditions may also arise from the choice of roughness parameter and its dimensional representation. This is particularly relevant to the present review, in which Ra predominated (21/27 studies), whereas alternative descriptors were less frequently reported.

The distinction between roughness parameters is important because they describe different statistical and dimensional properties of surface topography. Ra represents the arithmetical mean of the absolute profile height values, whereas Rq represents the root mean square of the profile height values and is therefore more sensitive to larger deviations. Rz describes the vertical distance between the maximum profile peak and valley and therefore captures a different aspect of surface topography from the average-height parameters Ra and Rq [55]. The corresponding areal parameters Sa, Sq, and Sz characterize analogous height features over an evaluation area rather than along a single profile [55,56]. This distinction is consistent with the standardized separation between profile surface-texture parameters and areal surface-texture parameters established in ISO 21920-2:2021 and ISO 25178-2:2021, respectively [57].

Corresponding 2D and 3D parameters may be strongly correlated but should not be considered numerically equivalent. In human enamel, Vieira et al. demonstrated significant positive correlations for Rz-Sz, Ra-Sa, and Rq-Sq, while also showing that these parameters originate from different linear and areal sampling representations [58]. Therefore, correlation between corresponding parameters does not justify direct substitution or pooling of their numerical values. This issue is particularly relevant to the present review because Ra dominated the included evidence, whereas only a small subset of studies reported additional descriptors; notably, Haberal and Çelik calculated Ra from three linear scans but Sa over the entire 2 × 2 mm scanned surface, illustrating that even values obtained from the same optical dataset represent different sampling domains [11]. Consequently, comparisons across studies should consider the specific roughness parameter, dimensional representation, sampling strategy, and measurement conditions rather than interpreting all reported roughness values as equivalent measures of enamel surface topography.

The subgroup analyses of profilometric methodology and dimensionality did not identify a consistent methodological advantage for either contact vs. non-contact/optical profilometry or profile-based 2D vs. areal/3D assessment. Although both contact and optical approaches were used to detect surface changes in experimentally demineralized and intervention-modified enamel, substantial methodological variability persisted within each subgroup, preventing the influence of measurement principle from being isolated. This limitation is relevant because direct comparative studies have demonstrated method-dependent differences between stylus and optical surface measurements [53,54].

Dimensionality was similarly difficult to evaluate independently, as several 3D-capable systems in the included studies reported profile-based Ra rather than areal parameters, indicating that instrument capability and reported outcome dimensionality are not synonymous. Accordingly, the present subgroup synthesis does not provide sufficient evidence to establish superior accuracy, validity, or reproducibility for either measurement principle or dimensional approach, as described in Table 3. Kranz et al. employed both optical and mechanical profilometry, although these approaches were applied to different dental substrates rather than providing a standardized head-to-head comparison on the same enamel surface [26]. Therefore, contact and optical profilometry should be regarded as methodologically distinct approaches, while their relative clinical applicability cannot be established from the current in vitro evidence.

A related concern was the incomplete reporting of profilometric procedures. Calibration or calibrated instrument status was explicitly mentioned in four studies, although the level of detail regarding the calibration procedure, reference standard, or frequency varied. Stylus/probe characteristics, scan dimensions, acquisition settings, filtering, and data-processing procedures were inconsistently documented. These methodological details are relevant because profilometric measurements are sensitive to several aspects of the measurement procedure. Acquisition conditions and instrument-related factors may affect measurement reliability, while scan dimensions and subsequent data-processing procedures, including leveling and background correction, can influence the resulting surface-roughness values. Therefore, incomplete reporting of these methodological characteristics may limit the reproducibility of profilometric assessments and complicate direct comparisons of surface-roughness outcomes across studies [59,60]. Consequently, incomplete reporting prevents full reconstruction of the measurement protocol and limits independent assessment of measurement reliability and reproducibility. Importantly, non-reporting does not indicate that calibration or appropriate acquisition procedures were not performed, nor that the resulting measurements were inaccurate. Rather, formal reproducibility cannot be established from the available evidence in the absence of sufficiently reported protocols and dedicated repeatability or reproducibility testing.

The present findings also require a distinction between the observed applicability of profilometry for experimental surface characterization and formally established measurement validity. Across the included studies, Aref and Alrasheed, Montaser et al., Guma et al., Pineda-Domínguez et al., Haberal and Çelik, and Özen et al. found that profilometry consistently quantified surface changes associated with experimental demineralization and subsequent interventions [11,23,24,29,31,42]. Although several included studies, such as studies by Kranz et al., Farooq et al., Pineda-Domínguez et al., Ahmed et al., Özen et al., and Naguib et al. additionally used SEM, Raman spectroscopy, micro-CT, microhardness, or elemental analyses [26,31,36,37,42,45], these techniques provided complementary morphological, compositional, subsurface, or mechanical information rather than direct validation of profilometric measurements. This distinction is supported by direct methodological comparisons showing that mechanical and optical measurement systems may yield different roughness values when evaluating the same surface [53,54]. These findings highlight the importance of considering the measurement principle and the specific profilometric approach when comparing surface-roughness outcomes across studies. In the present review, profilometry was consistently used to quantitatively characterize surface changes in experimentally demineralized enamel. However, the included studies did not provide sufficient formal validation or repeatability/reproducibility data to establish the measurement validity and reproducibility of profilometry specifically for this experimental context.

Complementary techniques provide different forms of evidence for interpreting profilometric findings because they assess distinct properties and spatial scales of the enamel substrate. SEM provides morphological visualization of surface alterations and may therefore support the interpretation of profilometrically detected topographical changes, but it does not independently validate numerical roughness values, as illustrated by its complementary use alongside profilometry in the included studies by Aref and Alrasheed, Guma et al., Barrera-Ortega et al., Aref and Alsdrani, Haberal and Çelik, Özen et al., and Naguib et al. [11,23,29,32,33,42,45]. Raman spectroscopy provides compositional and mineral-related information; in the study by Pineda-Domínguez et al. Profilometric Ra was assessed alongside phosphate-related Raman signals, providing parallel evidence of topographical and mineral changes rather than direct validation of roughness [31]. Micro-CT similarly provides complementary three-dimensional information on subsurface lesion characteristics, including lesion depth, surface area, volume, and mineral density [61], and may therefore indicate whether surface changes are accompanied by changes within the lesion body. AFM, although not used as a complementary technique in the included studies, can provide quantitative micro-/nanoscale topographical characterization; however, its different measurement scale and acquisition characteristics preclude assuming direct equivalence with profilometric roughness values [62]. Thus, concordance between profilometry and complementary techniques may strengthen the interpretation of associated morphological, topographical, compositional, or subsurface changes but should not be regarded as formal validation of profilometric roughness. Formal validation would require a standardized quantitative method comparison under comparable experimental conditions.

The recurrent RoBDEMAT concerns identified across the included studies may reduce confidence in the reported profilometric effects. Inadequate or insufficiently reported randomization may compromise group comparability, while inadequate sample-size justification can reduce confidence in the precision and credibility of the results [16]. The frequent absence or insufficient reporting of operator blinding may additionally increase susceptibility to measurement-related bias when measurement procedures involve operator judgment. Similar methodological shortcomings involving randomization, sample-size justification, and blinding were recently identified in a RoBDEMAT-based systematic review of laboratory studies assessing dental surface roughness by Esati et al. [63]. These concerns do not demonstrate that the reported profilometric measurements were inaccurate, but they reduce confidence in the robustness of the synthesized evidence and support a cautious interpretation of the reported effects.

Based on the methodological variability and recurrent reporting deficiencies identified across the included studies, we propose a minimum reporting framework for profilometric assessment of experimentally demineralized enamel and intervention-modified surfaces (Table 4). Future studies should report the profilometer manufacturer and model, measurement principle (contact or optical), dimensional representation (profile-based or areal), and calibration procedure and frequency. Acquisition parameters should be specified according to the measurement system, including stylus geometry, measuring force, traverse length, speed, and cut-off/filtering for contact profilometry, as well as scan area, lateral/vertical resolution, acquisition settings, and surface-processing procedures for optical systems. The sampling protocol should define the enamel measurement site, number and spatial distribution of traces or scans, repeated-measurement and averaging procedures, and experimental stage at assessment.

Finally, roughness outcomes should be reported using the appropriate profile- or area-based designation (e.g., Ra/Rq/Rz or Sa/Sq/Sz), together with measurement units and relevant data-processing procedures. This framework is intended as a minimum reporting proposal derived from the methodological gaps identified in the present review rather than as a formally validated reporting guideline.

Several limitations should be acknowledged. All included studies were conducted under in vitro conditions on extracted human teeth and artificially demineralized enamel surfaces, limiting direct extrapolation to naturally occurring clinical WSLs in the oral environment. Furthermore, restricting the search to studies published between 2021 and 2026 may have excluded earlier investigations that contributed important evidence to the methodological development and validation of profilometry. Accordingly, the present findings should be interpreted as an assessment of contemporary methodological practices rather than a comprehensive historical evaluation of profilometric methodology.

The restriction to English-language publications may also have resulted in the omission of relevant evidence published in other languages. In addition, although the search included multiple databases, relevant studies indexed exclusively in other databases may not have been identified. Together, these restrictions may have limited the completeness of the evidence base and should be considered when interpreting the findings of this review.

## 5. Conclusions

Profilometry appears to be a promising technique for quantitatively characterizing enamel surface topographical changes in experimentally demineralized and intervention-modified enamel as WSL models. However, the available evidence is characterized by substantial methodological heterogeneity, limited standardization of experimental and measurement protocols, incomplete reporting of key profilometric procedures, and a lack of formal validation and reproducibility testing. Consequently, the validity and reproducibility of profilometric assessment in these experimental models cannot yet be considered fully established. Post-treatment roughness values should therefore be interpreted as descriptors of modified surface topography rather than as direct indicators of lesion reversal or clinical treatment effectiveness. Furthermore, recurrent risk-of-bias concerns, particularly regarding randomization, operator blinding, and sample-size justification, further reduce confidence in the robustness of the synthesized evidence and support a cautious interpretation of the reported profilometric effects.

Greater methodological standardization, adoption of the proposed minimum reporting framework, and dedicated validation and reproducibility studies may improve the comparability and robustness of future research. Such methodological consolidation may also provide a stronger basis for subsequent translational and clinical studies evaluating the applicability of profilometric assessment to naturally occurring WSLs.

## Figures and Tables

**Table 1:** Summary of the characteristics of the included studies.

Author and Publication Year	Focus of Investigation	Sample Size	Results
Salih et al., 2025 [22]	Evaluation of the effect of MI Varnish™ (GC Corporation, Tokyo, Japan), fractional CO_2_ laser, and their combined application on demineralized enamel surfaces.	30 extracted human premolars (60 enamel specimens).	-Surface microhardness: *p* > 0.05. -SR: *p* > 0.05. -SEM: Smoother and more homogeneous enamel surfaces after all treatments, especially in the combined treatment groups. -EDX: Highest Ca wt% in sound enamel (44.3%); among treated groups, MI Varnish™ + CO_2_ laser showed the highest Ca wt% (39.0%).
Aref, Alrasheed, 2022 [23]	The focus of investigation is on the effectiveness of combining casein phosphopeptide amorphous calcium phosphate (CPP-ACP) with universal adhesive resin for treating WSLs, compared to other treatments like ICON (DMG Chemisch-Pharmazeutische Fabrik GmbH, Hamburg, Germany) resin and CPP-ACP alone. The study assesses color stability, surface microhardness, and SR.	45 extracted human premolars.	-Color difference: *p* < 0.05. -Surface microhardness: *p* < 0.05. -SR: *p* < 0.05.
Montaser et al., 2025 [24]	Comparison of the remineralization effectiveness of fluoridated toothpaste, Curasept (Curasept S.p.A., Saronno, Italy), and BioMin toothpaste (BioMin Technologies Ltd., London, UK) on artificial enamel lesions in primary teeth.	40 extracted human primary anterior teeth.	-Surface microhardness: *p* < 0.05. -SR: *p* < 0.05. -EDX: *p* < 0.05. -SEM: BioMin demonstrated the greatest surface remineralization with complete restoration of enamel morphology.
Khan et al., 2025 [25]	Comparison of the effects of resin infiltration (ICON), GC Fuji VII (GC Corporation, Tokyo, Japan), and Helioseal-F (Ivoclar Vivadent AG, Schaan, Liechtenstein) on artificial WSLs.	80 extracted human premolars.	-SR: *p* < 0.05. -Surface microhardness: *p* > 0.05. -Masking effect: *p* < 0.05.
Kranz et al., 2022 [26]	Evaluation of the remineralization potential of zinc-carbonate hydroxyapatite (biorepair^®^) toothpaste (Coswell S.p.A., Funo di Argelato, Bologna, Italy) on artificially demineralized human enamel and dentin.	40 extracted human third molars.	-Raman spectroscopy (enamel): *p* > 0.05. -Raman spectroscopy (dentin): *p* > 0.05. -Mechanical profilometry: *p* < 0.05. -EDX: calcium-phosphate and silicon deposits detected on treated dentin; no deposits detected on enamel. -White-light interferometry: enamel roughness unchanged (0.58 ± 0.02 vs. 0.58 ± 0.02 μm); dentin roughness 0.62 ± 0.16 vs. 0.51 ± 0.03 μm.
Alshahrani, Elrashid, 2024 [27]	Evaluation of the effect of different resin infiltration application modalities and bleaching on the SR of artificial WSLs.	96 extracted human premolars.	-SR: G1 and G4 (*p* < 0.05); G2 and G3 (*p* > 0.05). -Post hoc: G1 vs. G3 at T2 (*p* < 0.05); G2 vs. G4 and G3 vs. G4 at T3 (*p* < 0.05).
Mohamed et al., 2024 [28]	Comparison of the remineralizing effect of nano-silver fluoride (NSF) and 5% sodium fluoride varnish on artificial enamel caries-like lesions in permanent teeth.	15 extracted human molars and premolars (30 specimens).	-DIAGNOdent: baseline and after demineralization, *p* > 0.05; after treatment, *p* < 0.05. -SR: *p* < 0.05. Ca weight%: *p* < 0.05. P weight%: *p* < 0.05. -Ca/P ratio: *p* < 0.05.
Guma et al., 2023 [29]	Evaluation of the effects of sodium bicarbonate and erythritol air-polishing powders on the SR of sound and demineralized enamel during simulated orthodontic treatment.	42 extracted human caries-free molars.	-SR (sound enamel): *p* < 0.01. -SR (demineralized enamel, 5 s): *p* < 0.05. -SR (demineralized enamel, 150 s): *p* < 0.001.
Liu et al., 2024 [30]	Evaluation of the durability of resin infiltration on artificial WSLs after simulated long-term oral challenges (pH cycling, thermocycling, staining, and toothbrushing).	25 extracted human molars (100 enamel specimens).	-SR: *p* < 0.001. -Surface microhardness: *p* < 0.001 (pH cycling); *p* > 0.05 (thermocycling, staining). -Color difference (ΔE): *p* < 0.001 (pH cycling, staining); *p* < 0.05 (thermocycling); *p* > 0.05 (toothbrushing).
Pineda-Domínguez et al., 2025 [31]	Evaluation of the remineralizing effect of three fluorinated varnishes (Fluor Protector—Ivoclar Vivadent AG, Schaan, Liechtenstein, Clinpro White Varnish—3M ESPE, St. Paul, MN, USA, and Duraphat—Colgate-Palmolive, New York, NY, USA) on artificial enamel lesions using Raman spectroscopy, SR, fluoride release, and Vickers hardness.	75 extracted third molars (150 enamel surfaces; *n* = 30/group).	-Fluoride release (ISE-F): *p* < 0.05. -SR: FP (*p* = 0.0057; *p* = 0.0001); β-TCP (*p* = 0.0180; *p* = 0.0067); CDu (*p* = 0.0009; *p* = 0.0199). -Vickers microhardness: *p* < 0.05.
Haberal and Çelik, 2026 [11]	Evaluation of the effects of office and home bleaching agents containing nano-hydroxyapatite (n-HAP) on sound and demineralized enamel, assessing SR, microhardness, and enamel micromorphology.	120 enamel specimens obtained from 60 extracted human third molars.	-SR (Ra): *p* < 0.05. -SR (Sa): *p* > 0.05. -Surface microhardness: *p* < 0.05. -SEM: n-HAP groups exhibited mineral deposition and preservation of enamel morphology.
Barrera-Ortega et al., 2025 [32]	Comparison of β-TCP-F varnish and CPP-ACP-F paste on enamel remineralization, surface properties, and biofilm resistance.	120 third molar enamel specimens.	-Surface microhardness: *p* < 0.05. -SR: *p* < 0.05. -Wettability: *p* < 0.05. -Biofilm: *p* < 0.05.
Aref, Alsdrani, 2023 [33]	Evaluation of nano-hydroxyapatite-containing universal adhesive resin for the management of WSLs.	80 extracted permanent molars (160 specimens).	-Surface microhardness: *p* < 0.0001. -SR: *p* < 0.0001. -Color change: *p* < 0.0001.
Priyam et al., 2023 [34]	Comparison of the abrasiveness of three commercially available dentifrices on sound and demineralized enamel.	42 extracted human anterior teeth.	-SR: *p* = 0.005. -Intragroup comparison: Colgate (*p* = 0.018); Dant Kanti (*p* = 0.027); Glister (*p* > 0.05).
Tlaiye-García et al., 2025 [35]	Evaluation of CPP-ACP fluoride varnish on the roughness and surface morphology of deciduous enamel exposed to acidic beverages.	128 deciduous anterior teeth.	-SR: *p* < 0.05. -SEM: CPP-ACP fluoride varnish preserved enamel surface morphology, mainly after 4 days.
Farooq et al., 2021 [36]	Evaluation of the remineralization potential of fluoride-incorporated bioactive glass toothpaste compared with conventional fluoride toothpaste.	72 enamel blocks from maxillary first premolars.	-Surface microhardness: intragroup *p* < 0.05; intergroup *p* > 0.05. -SR: intragroup *p* < 0.05; intergroup *p* > 0.05. -Micro-CT: intragroup *p* > 0.05; intergroup *p* > 0.05.
Ahmed et al., 2025 [37]	Comparison of bioactive-glass-based experimental resin infiltrants regarding penetration, aging, and surface properties.	Human-extracted caries-free premolar teeth, 3 specimens per group.	-Microhardness: *p* > 0.05. -SR: *p* < 0.05. -Micro-CT: *p* < 0.05. -SEM: Micro-pits observed after thermal and chemical aging.
Ibrahim et al., 2023 [38]	Evaluation of repeated etching cycles during resin infiltration on demineralized enamel SR and esthetic outcomes.	90 extracted premolars.	-SR: *p* < 0.001. -Color change: *p* = 0.045; Resin 1 vs. Resin 2: *p* < 0.05. -Esthetics: Five etching cycles produced a color comparable to baseline.
Doğu Kaya et al., 2024 [39]	The focus of investigation is to evaluate the effect of using remineralization agents before resin infiltration on the treatment of initial enamel lesions, specifically assessing changes in surface properties such as microhardness and SR.	80 human molar enamel specimens.	-SR: *p* > 0.05 (between groups); *p* < 0.001 (treatment effect). -SR: *p* > 0.05. -DIAGNOdent Pen: *p* > 0.05 (between groups); *p* < 0.001 (treatment effect). -FluoreCam (size/intensity): *p* > 0.05 (between groups); *p* < 0.001 (treatment effect). -OCT: *p* > 0.05 (between groups); *p* < 0.001 (treatment effect). -Ultrasound: *p* > 0.05 (between groups); *p* < 0.001 (treatment effect). -SEM/EDX: Similar surface morphology among treatment groups; the lowest Ca (%atomic) was observed in the resin infiltration group.
Vahedi et al., 2022 [40]	Evaluation of the effect of different Er:YAG laser energy densities combined with fluoride varnish on the SR of demineralized enamel.	30 buccal and lingual slabs from impacted third molars.	-SR: FL6 vs. healthy enamel (*p* = 0.054); FL8 vs. healthy enamel (*p* = 0.027); fluoride varnish vs. healthy enamel (*p* = 0.029); FL24 vs. healthy enamel (*p* > 0.05).
Chabuk, Al-Shamma, 2023 [5]	Comparison of microabrasion (Opalustre™—Ultradent Products Inc., South Jordan, UT, USA), bioactive glass (Sylc^®^—Denfotex Research Ltd., Inverkeithing, UK), and resin infiltration (ICON^®^) for the treatment of enamel WSLs, evaluating changes before and after pH cycling.	75 extracted human permanent molars (100 specimens).	-SR: ICON vs. Opalustre and Sylc (*p* < 0.05); Opalustre vs. Sylc (*p* > 0.05). -Surface microhardness: ICON vs. Opalustre (*p* < 0.05); ICON vs. Sylc (*p* > 0.05); Opalustre vs. Sylc (*p* > 0.05).
Bolty et al., 2023 [41]	Evaluation and comparison of the remineralizing efficacy of chicken eggshell powder (CESP), propolis, and grape seed extract (GSE) on artificially induced enamel caries.	70 first premolars.	-Ca/P ratio: remineralization *p* = 0.93; after pH cycling *p* = 0.50. -Microhardness: remineralization *p* < 0.001; after pH cycling *p* < 0.001. -SR: remineralization *p* < 0.001; after pH cycling *p* < 0.001. -SEM/EDX: All remineralizing agents produced newly formed hydroxyapatite on demineralized enamel; twice-daily application resulted in greater mineral deposition and better resistance to pH cycling than once-daily application.
Inna et al., 2024 [4]	Evaluation of the staining susceptibility and SR of white-spot lesions (WSLs) treated with resin infiltration (RIT) and microabrasion (MA) after thermocycling in red wine.	78 extracted permanent premolars.	Both RIT and MA restored WSL color close to sound enamel. After thermocycling in red wine, RIT showed ΔE = 31.40 ± 4.89, while MA showed ΔE = 43.94 ± 3.57, with no significant difference between treatments in staining susceptibility (*p* > 0.05). -SR after thermocycling was lower for RIT (0.15 ± 0.03 μm) than MA (0.55 ± 0.09 μm), but the difference was not statistically significant (*p* > 0.05). -A moderate positive correlation was observed between color change and SR (Spearman rs = 0.577, *p* < 0.001), indicating that increased roughness was associated with greater staining.
Ozen et al., 2025 [42]	Evaluation of the effect of 5.25% NaOCl application before resin infiltration or fluoride-containing resin varnish on the treatment of WSLs.	160 human extracted premolars.	-Surface microhardness: *p* < 0.001. -SR: *p* < 0.001. -DIAGNOdent Pen: *p* < 0.001. -Micro-CT: *p* < 0.001; group × stage *p* > 0.05. -Microleakage: *p* < 0.001. -SEM: Resin-infiltrated and resin-varnish surfaces were smooth after treatment; aging induced surface irregularities, with microcracks predominantly in the resin varnish groups.
Alagha, 2026 [43]	Evaluation of the effect of brushing, bleaching, and polishing on the SR of ICON-treated enamel at different storage periods.	84 extracted anterior teeth.	-SR (treatment modalities): *p* < 0.05; Bleaching vs. Polishing: *p* = 0.0196; Control vs. Brushing: *p* > 0.05; Control vs. Polishing: *p* > 0.05; Brushing vs. Polishing: *p* > 0.05. -SR (storage period): *p* < 0.05; 1 day vs. 1 week: *p* > 0.05; 1 week vs. 1 month: *p* > 0.05
Khater et al., 2021 [44]	Evaluation of the effect of CPP-ACP, CPP-ACPF, and nano-hydroxyapatite on enamel remineralization during fixed orthodontic treatment.	120 specimens from extracted orthodontic patient teeth.	SR: -*p* < 0.05 (before vs. after demineralization/remineralization); -*p* > 0.05 (between remineralizing agents). Surface microhardness: -*p* < 0.05 (before vs. after demineralization/remineralization); -*p* > 0.05 (between remineralizing agents). -Color change: ΔE1 *p* = 0.26; ΔE2 *p* = 0.50; ΔE3 *p* = 0.28.
Naguib et al., 2025 [45]	Evaluation of the remineralization potential of magnesium oxide nanoparticles (MgO-NPs), alone and combined with conventional remineralizing agents, on artificial WSLs.	180 human molars.	-SR: *p* < 0.05; -MgO-NPs vs. other groups: *p* = 0.001. -Calcium deposition: *p* < 0.001; -GC/MgO vs. demineralized enamel: *p* < 0.05. -Phosphorus deposition: *p* < 0.01; -GC/MgO vs. demineralized enamel: *p* < 0.05. -Surface microhardness: 2 weeks: *p* < 0.05; 4 weeks: *p* = 0.03, 0.025, 0.020, 0.040, 0.024; 8 weeks: *p* = 0.028, 0.035, 0.016, 0.023, 0.024; between remineralized groups: *p* > 0.05. -SEM: Smooth enamel surface with mineral deposition after 8 weeks. -Raman spectroscopy: MgO-NPs showed the highest hydroxyapatite peak intensities after remineralization.

**Table 2:** Profilometric methods used and reported surface roughness parameters in the included studies.

Study	Instrument/Manufacturer	Measurement Principle	Dimensional Assessment Reported	Calibration	Scan area/Length and Key Acquisition Settings	Sampling/Measurement Protocol	Roughness Parameter(s) Reported
Salih et al., 2025 [22]	NR	NR	NR	NR	NR	SR assessed at R1 sound enamel, R2 demineralized enamel, R3 post-treatment.	SR (parameter not specified)
Aref and Alrasheed, 2022 [23]	Profilometer; Mitutoyo, Sakado, Japan	Contact stylus	Profile-based; Ra	NR	Accuracy 0.01 mm; cut-off 0.25–2 mm; traverse range 3 mm; diamond tip radius 2 μm; tip angle 60°; force 0.75 mN; velocity 0.5 m·s^−1^	Three measurements/specimen; averaged.	Ra (μm)
Montaser et al., 2025 [24]	MarSurf PS1; Mahr GmbH, Göttingen, Germany	Optical (as reported by authors)	Profile-based; Ra	No profilometer calibration procedure reported	Technical specifications NR	Four tracings perpendicular to enamel surface/specimen; averaged; measured after 28-day pH-cycling remineralization protocol.	Ra (μm)
Khan et al., 2025 [25]	NR	NR	NR	NR	NR	NR	SR (parameter not specified)
Kranz et al., 2022 [26]	Talysurf CCI HD, AMETEK Taylor Hobson Hobson Ltd., Leicester, UK; Hommel Tester T1000, Hommelwerke, GmbH, Villingen-Schwenningen, Germany	Optical non-contact white-light interferometry + contact stylus	Optical 3D acquisition with profile-based Ra; contact profile measurement	NR	Optical: 50×; field 330 × 330 μm; lateral resolution ~400 nm; vertical resolution sub-nm; measured area 0.55 × 0.55 mm (3 × 3 stitched fields); profile length 5 mm; Gaussian filter λc 0.8 mm. Contact: traverse 1500 μm; speed 0.15 mm/s.	5 measurements/specimen; 3 specimens/group for both systems.	Ra (μm)
Alshahrani and Elrashid, 2024 [27]	Contour GT; Bruker, Campbell, CA, USA	Optical non-contact	3D optical system; reported outcome designated only as SR	NR	Standard 5× objective; Vision64 v5.30; other technical specifications NR	Same predetermined region of interest; measurements after resin infiltration (T2) and after bleaching (T3).	SR (μm; Ra/Sa not specified)
Mohamed et al., 2024 [28]	Surftest 401; Mitutoyo, Kawasaki, Japan	Contact stylus	Dimensionality NR; SR parameter not explicitly named	NR	Stylus 5 μm pointer, 90°; constant speed 0.5 μm/s; force 4 μN	Three readings/specimen; mean recorded; assessed after treatment and 30-day pH cycling.	SR (unit/parameter not explicitly specified)
Guma et al., 2023 [29]	Cyberscan CT 100; cyberTECHNOLOGIES GmbH, Eching-Dietersheim, Germany	Non-contact confocal white-light sensor	2D profiles	NR	Vertical resolution 3 nm; probe z-resolution 0.02 μm; x/y step 1 μm; scan area 5 × 2 mm; five 5000-μm scan lines spaced 200 μm; Ra from five 4000-μm profiles	Measurements before/after treatment stages; five profiles used for Ra.	Ra (μm)
Liu et al., 2024 [30]	SuperView W1; CHOTEST, other manufacturer specifications NR	Non-contact optical	Profile-based; Ra; dimensionality otherwise NR	NR	Other acquisition specifications NR	Three repeated measurements/specimen; mean Ra; baseline and after simulated aging challenges.	Ra (nm)
Pineda-Domínguez et al., 2025 [31]	ZYGO Nexview 3D; Zygo, Middlefield, CT, USA	Non-contact optical	3D optical acquisition; profile-based Ra reported	NR	Representative 3D images at 100×; scanned area 83.139 × 83.139 μm^2^; objective specification, lateral/vertical resolution, cut-off, scan speed, filtering and software settings NR	Six random Ra measurements over 3 × 6 mm^2^ enamel surface; averaged; assessed at 0, 5, 10 and 15 days.	Ra (μm)
Haberal and Çelik, 2026 [11]	Profilm 3D; Filmetrics, San Diego, CA, USA	Non-contact optical, white-light interferometry	Both profile-based and areal	NR	Central 2 × 2 mm area; sensitivity 0.05 μm; ProfilmOnline software (KLA Corp., Milpitas, CA, USA), version of the software NR	Samples air-dried; Ra = average of three linear scans; Sa = entire scanned surface.	Ra and Sa (μm)
Barrera-Ortega et al., 2025 [32]	ZYGO 3D-Nexview; Zygo, Middlefield, CT, USA	Non-contact optical	3D optical system; profile-based Ra reported	NR	Objective lens, scan area, lateral/vertical resolution, cut-off, filtering and software settings NR	Measurements at baseline and after 5, 10, and 15 days of pH cycling.	Ra (μm)
Aref and Alsdrani, 2023 [33]	Surftest 211; Mitutoyo, Tokyo, Japan	Contact stylus	Profile-based; Ra	Calibrated with manufacturer’s standard calibration specimen before measurements	Distance 6 mm; speed 0.5 mm/s; force 0.75 mN; tip radius 2 μm; tip angle 60°	Five locations/specimen; mean Ra calculated.	Ra (μm)
Priyam et al., 2023 [34]	Surtronic S128; Taylor Hobson, UK	Contact stylus	Profile-based; Ra	Calibrated according to manufacturer’s instructions before measurements	Cut-off, stylus radius, traverse length, scan speed, force and resolution NR	Measured at baseline and after toothbrushing; average surface loss/Ra used.	Ra (μm)
Tlaiye-García et al., 2025 [35]	Surftest SJ-301; Mitutoyo, Tokyo, Japan	Contact stylus	Profile-based	NR	Diamond stylus; measuring modulus λ = 0.08 mm; speed 0.25 mm/s; traverse 3.0 mm; Gaussian filter	Three measurements at R0, R1 (4 days), R2 (7 days); mean calculated.	Ra and Rz (μm)
Farooq et al., 2021 [36]	Contour GT Optical Microscopes; Bruker, Tucson, AZ, USA	Non-contact optical	Profile-based; Ra	NR	Objective lens, scan area/length, lateral/vertical resolution, cut-off, filtering and software NR	Baseline, post-demineralization and post-remineralization; three scans/specimen; same predefined area; mean Ra.	Ra (nm)
Ahmed et al., 2025 [37]	Contour GT Surface Roughness Tester; Bruker Daltonics GmbH, Bremen, Germany	Non-contact optical	Profile-based; Ra	NR	Technical specifications NR	Before/after thermocycling and before/after 4-week chemical aging.	Ra (μm)
Ibrahim et al., 2023 [38]	Ambios XP-200; Ambios Technology, Inc., Milpitas, CA, USA	Contact stylus	Profile-based; Ra	NR	Vertical range 800 μm; scan-length range 50 mm; stylus radius 2.5 μm; actual scan 5 mm at 0.03 mm/s	Three measurements/specimen at six stages: baseline, post-etching, Resin 1, Resin 2, 7 days, 28 days.	Ra (μm)
Doğu Kaya et al., 2024 [39]	M300C; Mahr, Germany	Contact	Profile-based; Ra	NR	Stylus radius, cut-off, scan length, traverse speed, force, software, and resolution NR	Baseline, post-demineralization and post-treatment.	Ra (μm)
Vahedi et al., 2022 [40]	TR200; Time Group Inc., Beijing, China	Contact stylus	Profile-based	NR	Technical specifications NR	Measured after pH-cycling protocol and after experimental treatments.	Ra, Rz, Rpmyz
Chabuk and Al-Shamma, 2023 [5]	Leeb 432A; Leeb Instrument Co. Ltd., Chongqing, China	Contact stylus/mechanical 2D	2D profile-based	NR	Diamond stylus 5 μm tip diameter; traverse 1.25 mm; cut-off 0.25 mm; force <0.004 N	Mean of three readings/specimen; baseline, post-demineralization, post-treatment, and post-pH cycling.	Ra (μm)
Bolty et al., 2023 [41]	Proscan 2000; Scantron, Taunton, England	Non-contact optical	3D optical system; reported parameter not specified	NR	ZYGO Maxim-GP 200 software; other technical specifications NR	Baseline, post-demineralization, post-remineralization, and post-pH cycling.	SR (μm; parameter not specified)
Inna et al., 2024 [4]	InfiniteFocus G5; Alicona, Raaba/Graz, Austria	Non-contact optical	Profile-based Ra reported; dimensionality otherwise NR	NR	Measurement site: mid-buccal enamel; other technical specifications NR	Baseline, after artificial WSL formation, post-treatment, and after artificial-saliva storage or red-wine thermocycling.	Ra (μm)
Özen et al., 2025 [42]	MarSurf PS10; Mahr, Göttingen, Germany	Contact	2D profile-based	Performed after every three measurements	Cut-off 1.5 mm; diamond tip radius 2.0 μm; force 0.7 mN; speed 1.0 mm/s	Baseline, post-demineralization, post-treatment, and after 5000-cycle thermocycling.	Ra
Alagha, 2026 [43]	Surf-Corder mod. 1700; Kosaka, Tokyo, Japan	Non-contact	Profile-based roughness parameter; dimensionality otherwise NR	NR	Magnification ×20; cut-off 0.8 mm	After ICON and treatment modality; assessed at 1 day, 1 week, and 1 month in artificial saliva.	Ry (μm)
Khater et al., 2021 [44]	DEKTAK-3 v2.13; manufacturer not clearly reported (article states Uberingen, Germany)	Contact stylus	Profile-based; Ra	NR	Diamond stylus tip radius reported as 2 Nm by authors; speed 0.25 mm/s; cut-off 0.8 mm	Before pH cycling, after pH cycling, and after remineralization; average Ra/specimen.	Ra (μm)
Naguib et al., 2025 [45]	Profilometer; Nanovea Inc., SC, USA, city NR	Contact	Contact-mode 3D image acquisition; Ra reported	NR	Cantilever-mounted probe 3 μm; spring constant 0.9 N/m; 3D images; 256 × 256 pixel resolution	Before demineralization, after demineralization, and after remineralization at 2, 4, and 8 weeks.	Ra

**Table 3:** Methodological comparison of contact and non-contact/optical profilometry for surface roughness assessment in experimentally demineralized enamel as a WSL model in included studies.

Aspect	Contact Profilometry	Non-Contact/Optical Profilometry	Interpretation Based on the Available Evidence
Measurement principle	A stylus mechanically traces the surface, generally generating profile-based data	Surface height is acquired optically without mechanical contact; systems may generate profile-based or areal data	The approaches use different surface-sampling mechanisms and should not be considered directly interchangeable
Potential advantages	Widely used; relatively accessible; established profile-based roughness assessment	Avoids mechanical interaction with the surface; permits broader surface mapping and potential 3D areal characterization	These are general methodological characteristics; the included studies did not directly compare their practical performance under standardized conditions
Main limitations	Stylus geometry may prevent access to narrow valleys; measuring force may affect fragile surfaces; results depend on traverse length, speed, cut-off, and filtering	Results may be influenced by optical principle, surface reflectivity, scan area, resolution, acquisition conditions, missing-data treatment, filtering, and surface processing	Both methods are sensitive to instrument- and protocol-related factors
Reported parameters	Predominantly profile-based parameters such as Ra, Rq, Rz, Ry	Profile-based or areal parameters, including Ra/Rq/Rz/Ry and Sa/Sq/Sz/Sy	Instrument capability and reported outcome dimensionality are not synonymous; a 3D-capable instrument may still report Ra
Accuracy and reproducibility	Dependent on calibration, stylus characteristics, acquisition settings, sampling strategy, and data processing	Dependent on calibration, optical characteristics, resolution, acquisition settings, sampling strategy, and data processing	The included studies did not provide sufficient head-to-head evidence to establish superior accuracy or reproducibility for either approach
Applicability to experimental enamel	Used to detect surface changes in experimentally demineralized and treatment-modified enamel	Used to detect surface changes in experimentally demineralized and treatment-modified enamel	Both approaches were applicable under in vitro experimental conditions
Clinical applicability	Not established by the included evidence	Not established by the included evidence	Direct clinical applicability cannot be determined from exclusively in vitro studies

**Table 4:** Proposed minimum reporting framework for profilometric assessment of experimentally demineralized and intervention-modified enamel surfaces.

Reporting Domain	Minimum Information to Report
Instrument and measurement principle	Manufacturer and model; contact or non-contact/optical measurement principle; profile-based (2D) or areal (3D) assessment
Calibration	Calibration procedure and reference standard; calibration frequency
Contact profilometry settings	Stylus geometry/tip radius; measuring force; traverse/evaluation length; measurement speed; cut-off and filtering settings
Optical profilometry settings	Scan area/dimensions; lateral and vertical resolution, where applicable; acquisition settings; filtering and surface-processing procedures
Sampling and measurement protocol	Enamel surface/site assessed; measurement location and orientation; number and spatial distribution of traces/scans; repeated measurements; averaging procedure
Experimental stage	Clearly defined measurement time point(s), e.g., baseline, post-demineralization, post-intervention, and post-aging/challenge
Roughness outcomes	Parameter(s) with appropriate profile/areal designation (e.g., Ra, Rq, Rz vs. Sa, Sq, Sz); measurement units
Data processing	Software and version, where relevant; leveling/form removal; filtering; parameter calculation; and aggregation procedures

## Data Availability

No new data were created or analyzed in this study. Data sharing is not applicable to this article.

## Supplementary Materials

The following supporting information can be downloaded at: https://www.mdpi.com/article/10.3390/jfb17090429/s1, Supplementary Table S1. PRISMA 2020 main and abstracts checklists.

## Abbreviations

The following abbreviations are used in this manuscript:

WSL(s)	White Spot Lesion(s)
Ra	Arithmetic Mean Roughness
RI	Refractive Index/Refractive Indices
AFM	Atomic Force Microscopy
SEM	Scanning Electron Microscopy
CPP-ACP	Casein Phosphopeptide-Amorphous Calcium Phosphate
PICO(S)	Population, Intervention, Comparator, Outcome, and Study Design
2D	Two-Dimensional
3D	Three-Dimensional
Rq	Root Mean Square Roughness
Rz	Maximum Height of the Profile
Sa	Arithmetical Mean Height of the Surface
Sq	Root Mean Square Height of the Surface
Sz	Maximum Height of the Surface
MEDLINE	Medical Literature Analysis and Retrieval System Online
micro-CT	Micro-Computed Tomography
RoBDEMAT	Risk of Bias in Dental Materials
NR	Not Reported
PRISMA	Preferred Reporting Items for Systematic Reviews and Meta-Analyses
SR	Surface Roughness
EDX	Energy-Dispersive X-ray Spectroscopy
NSF	Nanosilver Fluoride
F-ISE	Fluoride Ion-Selective Electrode
n-HAP	Nano-Hydroxyapatite
β-TCP-F	Fluoride-Containing Beta-Tricalcium Phosphate
CPP-ACP-F	Casein Phosphopeptide-Amorphous Calcium Phosphate Fluoride
CESP	Chicken Eggshell Powder
GSE	Grape Seed Extract
RIT	Resin Infiltration Treatment
MA	Microabrasion
ΔE	Color Difference
MgO-NPs	Magnesium Oxide Nanoparticles
TEGDMA	Triethylene Glycol Dimethacrylate
UDMA	Urethane Dimethacrylate
ISO	International Organization for Standardization
OSF	Open Science Framework
AI	Artificial Intelligence
